# Identification of natural and anthropogenic signals in controlled source seismic experiments

**DOI:** 10.1038/s41598-022-07028-3

**Published:** 2022-02-24

**Authors:** J. Díaz, I. DeFelipe, M. Ruiz, J. Andrés, P. Ayarza, R. Carbonell

**Affiliations:** 1 Geosciences Barcelona, Geo3Bcn CSIC, c/ Solé Sabarís s/n, 08028 Barcelona, Spain; 2 Department of Geology, Universidad de Salamanca, Plaza de la Merced, s/n, 37008 Salamanca, Spain; 3 Institut Cartogràfic i Geològic de Catalunya, c/ Parc Montjuic s/n, 08038, Barcelona, Spain

**Keywords:** Atmospheric dynamics, Civil engineering, Seismology

## Abstract

The analysis of the background noise in seismic networks has proved to be a powerful tool not only to acquire new insights on the crustal structure, but also to monitor different natural and anthropogenic processes. We show that data acquired during controlled source experiments can also be a valuable tool to monitor such processes, in particular when using high-density deployments. Data from a wide-angle reflection and refraction seismic profile in the central-northwest part of Iberia is used to identify signals related to aircrafts, road traffic, quarry blasts, wind blow, rainfall or thunders. The most prominent observations are those generated by a helicopter and an airplane flying following trajectories subparallel to the profile, which are tracked along 200 km with a spatial resolution of 350 m, hence providing an exceptional dataset. Other highlights are the observation of the Doppler effect on signals generated by moving cars and the high-density recording of acoustic waves generated by thunders. In addition to the intrinsic interest of identifying such signals, this contribution proves that it is worth inspecting the data acquired during seismic experiments beyond the time interval including the arrival of the seismic waves generated by the controlled source.

## Introduction

In recent years, an increasing number of controlled source seismic experiments are recorded using a large number of light autonomous dataloggers, usually referred to as “nodes”, allowing a dense sampling of the investigated area, with interstation distances ranging from hundreds of meters to few kilometers. Each of these instruments is equipped with independent storage and timing system (GPS receiver or internal clock) and hence, contrarily to typical multichannel seismic systems, provide independent records similar to those recorded by classical dataloggers.

Complementary, the study of the vibrations recorded in absence of earthquakes, usually referred to as “seismic noise”, has boosted during the last decade, in particular with the development of ambient noise tomography. It has been proved that the seismic noise can be useful to monitor human activities^1,2^ or non-tectonic natural processes^3,4^. This has led to the development of the so-called environmental seismology, focused on the use of seismic data to investigate natural processes like river flow, landslides, rockfalls, ocean waves etc.

The first step of signal processing in controlled source surveys is to select the time window of interest for each trace according to its distance to the source, discarding the rest of the recorded signal. Typically, nodes are programmed to record from few minutes to some hours after each of the time windows where sources are planned to be set off. Therefore, in many experiments there is a significant amount of data which is not usually explored. The objective of this contribution is to evidence that this supplementary data includes vibrations generated by multiple natural and human-related sources which can be of interest in different research fields. We use data from a wide-angle reflection and refraction deep seismic profile in central-north west Spain to show that the identification and analysis of seismic signals can be useful to monitor very different processes, from meteorological events (rainfall, thunders, wind) to road traffic and aircraft activity. This work represents a clear example of the potential of reusing seismic data following the FAIR (findable, accessible, interoperable and reusable) principles of data management^5,6^.

## Data set

**Figure 1:**
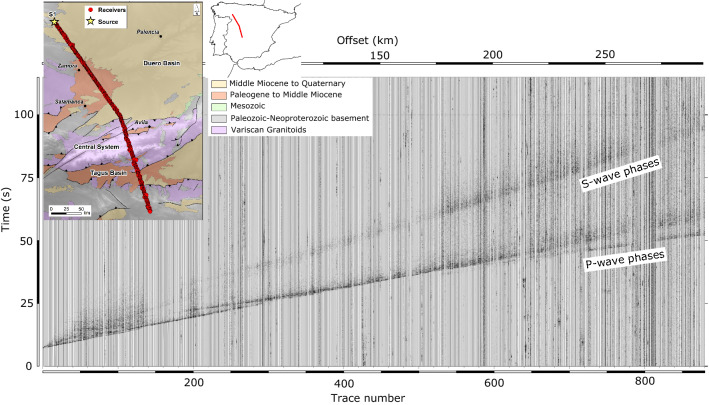
Vertical component record of the S1 shot along the CIMDEF profile, represented without time reduction after applying a 1–15 Hz band-pass filtering. Different P-wave phases can be identified even at this large scale. S-waves are also identified all along the profile. The insets show a simplified geological map of the sampled zone and its location in the Iberian Peninsula. This map has been produced using ArcGis 10.5 (https://www.esri.com/en-us/arcgis/products/arcgis-desktop/). The geological features are from the Geological Survey of Spain (IGME)^14^ and the topography is from CIAT-CSI SRTM (http://srtm.csi.cgiar.org)^15^.

The data analyzed here were acquired in July 2019 as part of CIMDEF project (“Central Iberian Massif DEFormation mechanisms”). This project aims to obtain a model of the crust and upper mantle structure along the Central System and its foreland basins: the Duero and Tagus Cenozoic basins (Fig. 1). The CIMDEF seismic profile^7,8^ is approximately 330 km long and runs along a NW–SE direction crossing the Tagus Basin, the Central System and the Duero Basin, extending northwards of the previously acquired ALCUDIA and IBERSEIS wide-angle transects^9,10^. The project included different deployments, some of them aiming to record earthquakes and seismic ambient noise^11,12^. A first deployment, carried out in 2017 and the 2019 deployment analyzed in this contribution were designed to record wide-angle reflections and refractions from controlled explosions fired at the northern and central part of the profile. These were registered by up to 996 Sercel RAU nodes equipped with 10 Hz geophones, with a station spacing close to 350 m. Stations were programmed to record during five hours (11:00–16:00 GMT) using a sampling rate of 250 samples per second. We will focus here on the data acquired on July 3rd, 2019, corresponding to the S1 shot, fired at the northern termination of the CIMDEF profile. Figure 1 shows a record section of this shot, clearly displaying P and S waves all along the profile. A first ray-tracing model of this shot is included in^13^. The reader is referred to this paper to get the details of its tectonic interpretation.

During the quality control of the raw data, we observed strong signals recorded several minutes after the P and S waves generated by the S1 shot. These signals feature amplitudes larger than the shot records, propagate south to north and are visible along more than 200 km in the raw data. This motivated us to explore the rest of the recording, which led us to observe a second highly energetic phase arriving about two hours later and propagating, in this case, from north to south. The apparent velocity observed for these phases, close to 200 km/h for the first one and to 600 km/h for the second case, together with their spectral content (see discussion below) strongly suggest that they are generated by aircrafts moving along the seismic profile.

**Figure 2:**
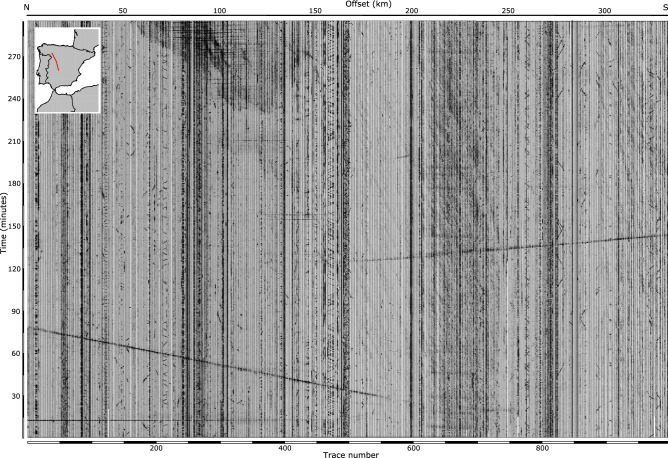
Raw seismic data acquired on July 3rd, 2019 along the CIMDEF profile. The time scale covers an interval of 5 h. Traces are displayed using a common amplitude scale. The lower horizontal scale refers to trace numbers. The distance to the northern end of the profile is shown by the upper horizontal scale. The inset map shows the geographic location of the profile. The S1 shot signal (Fig. 1) appears here as a subhorizontal line arriving approximately at 12 min.

Figure 2 shows the complete five hours of data recorded at the 996 receivers deployed along the profile. The image is dominated by these two signals, and is somehow similar to wide-angle reflection and refraction record sections, but having a much larger time scale. The image includes plenty of other signals, some of them affecting just a few traces and lasting few seconds, while others appear over large zones and lasting several minutes. Next, we will discuss the origin of the most relevant among the identified signals, related to wind and thunders, rain, quarry blasts, road and train traffic or aircrafts overflights.

## Signals generated by anthropogenic sources

### Helicopter signals

**Figure 3:**
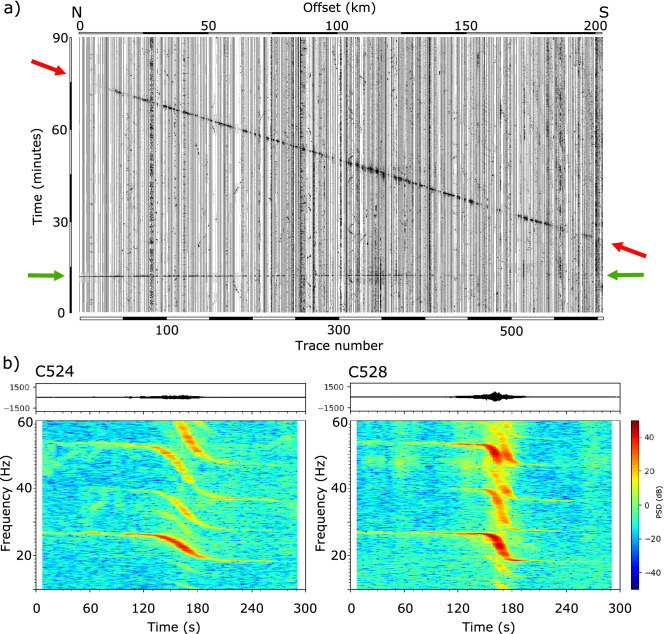
(**a**) Record section showing the signals generated by the CIMDEF controlled source (green arrows) and by a helicopter overflying the profile (red arrows). (**b**) Raw signals and spectrograms obtained for stations C524 and C528 during the time interval corresponding to the overflight of a helicopter.

The most prominent anomalous arrival is observed along more than 200 km in the northern part of the CIMDEF profile. The signal is first identified around trace C550 30 min after the beginning of our records. This phase can be followed to the northern edge of the profile, where it arrives 45 min later (Fig. 3a). The amplitude of the signal is strong and it can be identified without any kind of signal processing. Its apparent velocity, close to 200 km/h, suggests that it can be related to the acoustic waves generated by an aircraft moving along a SE to NW direction subparallel to the profile. The seismic records will then correspond to the coupling of acoustic waves to seismic waves in the vicinity of each seismic node.

Figure 3b shows two examples of the signal and the corresponding spectrogram. The most relevant features in the spectrograms are (i) the presence of a large number of harmonics and (ii) the gliding of each of the harmonics between a maximum and a minimum value. These signals can be identified in the seismograms during 4–5 min. The presence of an elevated number of harmonics with gliding spectra is a well-known feature associated with helicopters overflying seismic stations and are mostly generated by the blades of the main and tail rotors^16–18^. Depending on factors as topography, wind direction or wind speed, helicopters can be seismically detected at distances of up to 40 km from the recording site. The characteristics of the signal depend strongly on the so-called blade-passing frequency (BPF), equivalent to the shaft frequency times the number of blades. For helicopters with four blades, the fundamental frequency of the main rotor usually ranges between 8 and 26.8 Hz and has several harmonics^16^. The tail rotor is usually a multiple of the main rotor frequency.

The observed frequency gliding (Fig. 3b) is due to the Doppler effect, generated when a moving source passes over a stationary receiver. The relative motion between the moving sources and the receivers results in an alteration of the observed frequency, which appear different from its real value. This effect is widely observed in either acoustic, elastic or electromagnetic waves and has been used for many different applications, from the speed radars used by police to astronomical or medical uses, as it allows to estimate the velocity of the moving source. In our case, we have used the Doppler effect formula (see “Methods” section) to estimate the velocity value for 25 stations distributed along the profile, obtaining an average value of 183 km/h with a standard deviation of 8.1 km/h.

Eibl et al.^19^ have shown that it is possible to invert the frequency-time curves for each receiver to deduce not only the velocity of the source, but also the source-receiver distance. Changes in the slope of the gliding signal reflects variations in the source-receiver distance, while changes in the frequency range of the signal shows variations of the velocity of the moving source. In the examples presented in Fig. 3, corresponding to stations located 1.4 km apart, the slope of the gliding signal clearly differs, reflecting variations in the source-receiver distance. We have estimated source-receiver distances for a selected group of stations by calculating their synthetic frequency/time curves and comparing them with the data. In all the investigated cases, the source remains close to the profile, with distances not exceeding 1.5–2.0 km. As an example, the source-receiver distances estimated for the two traces shown in Fig. 3 are of 1000 m for C524 and 300 m for C528. Despite these variations, it is important to note that the observation of a clear and overall similar Doppler signature all along the profile proves that the helicopter followed a path subparallel to the CIMDEF profile along more than 200 km.

We must highlight that, although recording waves generated by helicopters is quite usual in permanent and semi-permanent seismic networks, the present data allowed to track the aircraft along a journey of more than 200 km between central and northwestern Spain with a lateral resolution of 350 m, which is really exceptional.

### Airplane signals

**Figure 4:**
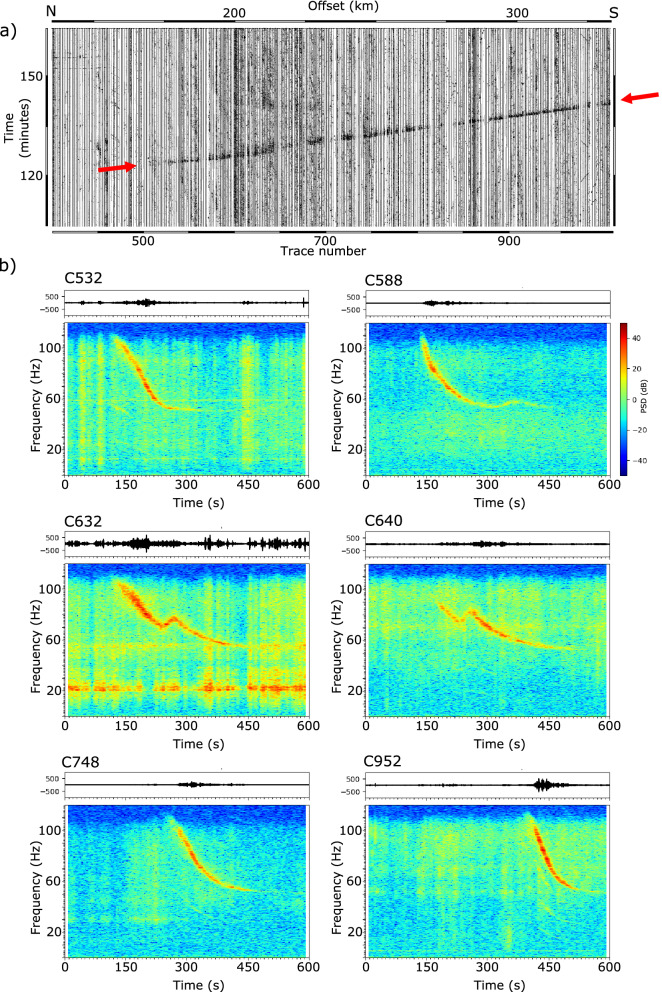
(**a**) Record section showing the arrival of the signals generated by an airplane overflying the profile (red arrows). (**b**) Raw signals and spectrograms for selected stations recording the overflight of the airplane. Variations in the shape of the spectrograms are interpreted as resulting from changes in the airplane trajectory (see text).

The second anomalous signal observed in our dataset starts at offsets of around 175 km (trace C510) and can be followed up to the southern termination of the profile, more than 150 km away. It is firstly observed 125 min after the zero time in the center of the profile and 140 min after the zero time at the southeastern edge of the profile (Fig. 4a). The apparent velocity of this signal is close to 600 km/h, clearly higher than that of the helicopter records. In this case, the source moves along a direction subparallel to the profile, but following an almost opposite sense than in the previous case. Spectrograms are also clearly different from the previous ones, as no harmonics are now observed (Fig. 4b). The high apparent velocity and the absence of harmonics point out to an origin related to an airplane overflying the stations^20^.

Spectrograms of the airplane signal show, as in the case of the helicopter signals, a clear Doppler effect. However, the highest frequency value cannot be observed, as it is above 120 Hz, the uppermost frequency that can be investigated with our data. For most of the traces, the frequency of the acoustic source is estimated in 80 Hz and the velocity calculated from the Doppler effect is close to 670 km/h. Between traces C580-C660 the spectrograms show an alteration, as the gliding seems to split into two branches by a frequency peak moving in time (Fig. 4b, middle panels). As noted by^19^, changes in the trajectory and/or velocity of a flying aircraft result in complex spectrograms. Therefore, this anomaly can be related to a change in the flying path, although other factors, as the influence of topography, should be discussed. Later on, from trace C660 to the end of the profile, the gliding signals are again similar to those in the northern section. A plausible departing point of this airplane is the Salamanca airport (Fig. 1), located 30 km to the west of the CIMDEF profile, at the same latitude as trace C480. Since 2015, this airport is not offering regular commercial flights. However, it is used for logistics and training activities and it serves as a base of the Spanish Armed Force, and also hosts firefighting services of the Spanish government. Therefore, we can roughly estimate that after taking off from the Salamanca airport, the plane moved to the southwest, changed its heading near the city of Ávila (traces C580–C660) and finally followed a direction subparallel to the CIMDEF profile.

Between traces C620 and C710, the data show a series of coherent arrivals appearing as subhorizontal large amplitude bands, separated by time intervals ranging from few minutes to more than one hour (Fig. 2). The signals do not have a clear onset and last 2–3 min. Their duration, waveform and spectral content suggest that they may correspond to commercial airplanes approaching or leaving the Adolfo Suárez-Madrid Barajas international airport, located 135 km to the east of the profile. Trace C640 is located in the vertical projection of one of the major air tracks followed by airplanes leaving the Madrid-Barajas airport (BARDI enroute, http://insignia.enaire.es, last access 23 December 2021), supporting hence this interpretation.

### Road traffic

As for the case of trains^21,22^, the use of car traffic as a seismic source to image the uppermost crust is an active research field. The most recent contributions in this topic use data recorded by high-density node networks^23–26^ or Distributed Acoustic System (DAS) sensors^27–29^ and are providing valuable results related to the subsurface structure.

**Figure 5:**
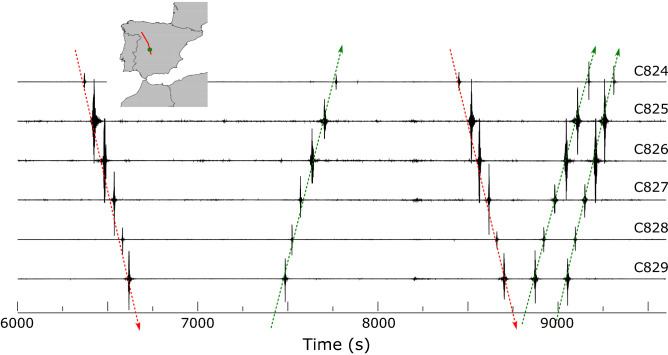
Seismic record of the waves generated by five vehicles moving along the seismic profile during a 40–50 min interval (traces C824-C829). Red lines mark vehicles moving southward and green lines those moving northward. The recording stations were installed along an unpaved road giving access to mountain areas and therefore, the low apparent velocities observed, ranging between 23 and 30 km/h seem reasonable.

The inspection of the data in Fig. 2 shows a large number of signals that are related to moving vehicles along roads located close to the seismic profile. This kind of signals appear as short and high amplitude spikes at a limited number of neighbor seismic stations (e.g. around traces C220, C470, C780 or C820). Figure 5 shows an example of seismic signals related to vehicles moving with apparent velocities below 30 km/h. In most cases, this kind of signals have higher apparent velocities, usually between 40 and 80 km/h. It must be noted that these apparent velocities can differ significantly from the real ones due to the relative position between the roads and the seismic profile. In case of roads crossing perpendicularly the profile, only one or two sites will record the signal while for roads subparallel to the seismic line, each vehicle can be followed along a large number of stations (see, for example, traces C460–C480 in Fig. 2).

**Figure 6:**
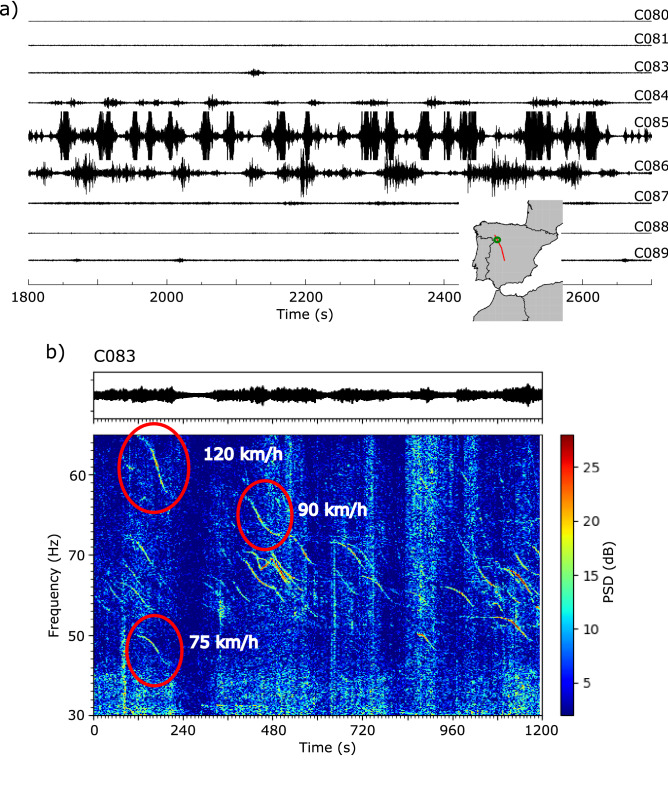
(**a**) 30 min of raw data recorded in stations C080-C089, located close to the A52 highway (green circle in the inset map). Traces are represented at true amplitude. Trace C085 is located close to the highway, while the distances between the later and traces C080 and C089 are 1.8 and 1.5 km respectively. (**b**) Raw signal and corresponding spectrogram as recorded at station C083, located at 750 m of the highway. Representative events are marked with circles and labeled with the velocities calculated using the Doppler formula.

A second kind of seismic record associated with road traffic appears when the seismic line crosses major highways. In this case, the seismic traces close to the highways have a high number of individual events, appearing in the global seismic record as groups of large amplitude, blackish zones. Some examples can be observed in Fig. 2 around traces C080 (A52 highway), C400 (A62 highway) and C490 (A50 highway). Figure 6a displays 9 traces located close to the intersection point with the A52 highway, connecting northwest and central Spain. There is a large difference in amplitudes between the sites close to the highway (e.g. C085) and those located at distances not exceeding 1.8 km (e.g. C080).

Figure 6b shows the raw signal and the corresponding spectrogram of an interval of 20 min recorded at station C083, located at 750 m from the A52 highway. Although signals associated with individual cars are difficult to discern in the seismogram, they can be easily separated and identified in the spectrogram, where they appear as gliding features similar to those generated by aircrafts and discussed previously. Doppler effects on seismic data due to passing trains have been described in recent years^21,30^ but observing this effect on cars is, to our knowledge, unreported. Applying the Doppler equation with a media velocity of 345 m/s, realistic velocities ranging between 75 and 120 km/h are calculated. We interpret that, similarly to what happens with aircraft related signals, the origin of these gliding spectra is the acoustic (pressure) air waves generated by moving cars that couples to the ground in the vicinity of the seismometers. Needless to say, this observation opens the door to the eventual use of geophones as a tool for traffic speed monitoring.

### Quarry blasts

**Figure 7:**
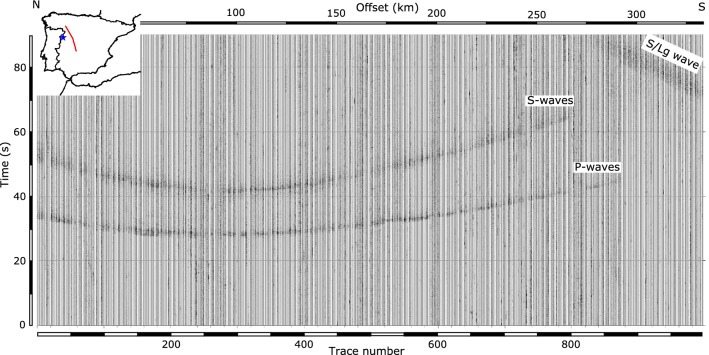
Quarry blast record. The inset maps show the procedure to locate the source of the explosion, a tungsten mine near the Portugal border. The blue star shows the position of the located source.

The inspection of this dataset has also allowed us to identify a high amplitude signal arriving at around 13:25 UTC, best observed in the northern part of the profile. Band-pass filtering between 1 and 15 Hz enhances the signal, thus being observed along more than 300 km (Fig. 7), similarly to the signal generated by the CIMDEF explosive source. Two energy packets are clearly identified, with a minimum time difference of 12 s around trace C275. At the northern end of the profile, the time difference between these phases is close to 18 s, while to the south the difference is around 30 s. The characteristics of this signal strongly points out to a seismic source generating P and S waves and located offline the CIMDEF profile, hence resulting in a record section with a fan geometry.

In order to locate this source, we have picked 263 P and S-phase time arrivals all along the profile and used Hypo71, a standard seismological procedure to locate earthquakes (see “Methods” section). The obtained epicenter (41.051°N, 6.665°W) is located near the Portugal border, at 100 km of the C280 trace, the most proximal point of the profile. The epicentral zone host the Barruecopardo Mining and Processing site, operated by the Saloro company (https://areaclientes.maclucan.com/saloro/en/barruecopardo-en/overview/, last accessed 23 December 2021). This open-pit mine produces high quality tungsten and its production was retaken in early 2019 after several decades without operation.

It seems straightforward to relate the signal identified in the seismic data to a blast related to the open-pit mining operations at the Barruecopardo mine. The analysis of seismic waves generated by this source can provide relevant information, as the acquisition of seismic profiles with fan geometry has proved to be useful to detect lateral variations in the crustal structure^31,32^. Modeling of this fan profile can provide additional constraints to the crustal structure deduced from forward 2D ray-tracing modeling along the CIMDEF line^13^.

As noted in Fig. 7, the waves of this quarry blast at the southern part of the profile are followed by a large band of energy arriving 70 s after our zero time. We have identified this feature as corresponding to the S/Lg waves generated by a small earthquake (magnitude 1.8 IGN) with epicenter in southern Spain, at 175 km to the south of the CIMDEF profile. Although the signals do not seem to be clear enough to provide valuable constraints, this observation helps to keep in mind that natural earthquakes can be recorded in datasets geared for controlled source experiments.

## Signals generated by natural weather-related sources

### Wind and wind turbines

**Figure 8:**
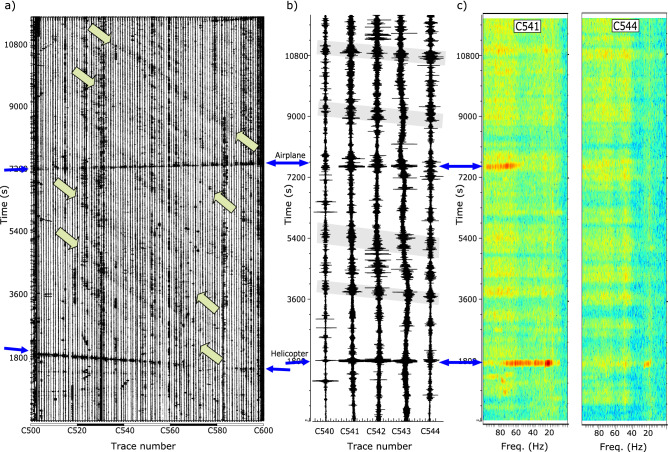
(**a**) Raw data for stations C500-C600, including the helicopter and airplane signals discussed in previous sections (thin blue arrows). Thick green arrows mark some examples of the wind gusts. (**b**) Subset of traces C540–C544 showing with grey boxes some of the wind gusts. (**c**) Spectrograms for traces C541 and C544), separated by 1.3 km. Intervals with blowing winds appear as bands of increased energy, better identified at frequencies above 20–40 Hz (color scale as in Figs. 3 and 4).

The movements of the vegetation and the atmospheric pressure variations induced by blowing winds result in an increase of the amplitude of the seismic background signal^33–35^. Wind generated vibrations are however difficult to isolate, as their dominant frequency, typically below 10–20 Hz, is also affected by anthropogenic noise. The record section in Fig. 2 shows correlated patches of relatively high amplitude appearing as steep bands between traces C500–C700. A more detailed view of these signals is presented in Fig. 8a, where traces C500–C600 are shown. The wave-trains last 5–10 min and have apparent velocities of 20–30 km/h. Figure 8b shows a blow-up of 5 of these traces that evidences the clear correlation between them, regardless of some differences in the respective waveforms. In the spectrograms (Fig. 8c) the signals appear as high energy bands extending across the spectra, but better identified for frequencies above 20–40 Hz. The spectrograms for the two selected sites, located just 1300 m apart, show that the energy packets related to wind blows arrive with a time difference of around 3 min, confirming an apparent velocity close to 25 km/h.

The duration of the signals, its apparent velocity and its spectral content strongly suggest an origin related to intermittent wind gusts. Note that the area where these signals are identified corresponds to the stations located across the Central System. The slope defined by the arrival times points to a wind blowing from the south/southeast. Consistently, the record section in Fig. 2 shows that the mean amplitude of traces C600-C700 is clearly higher than for that of stations C500-C600, located in the less windy northern side of the mountain range.

**Figure 9:**
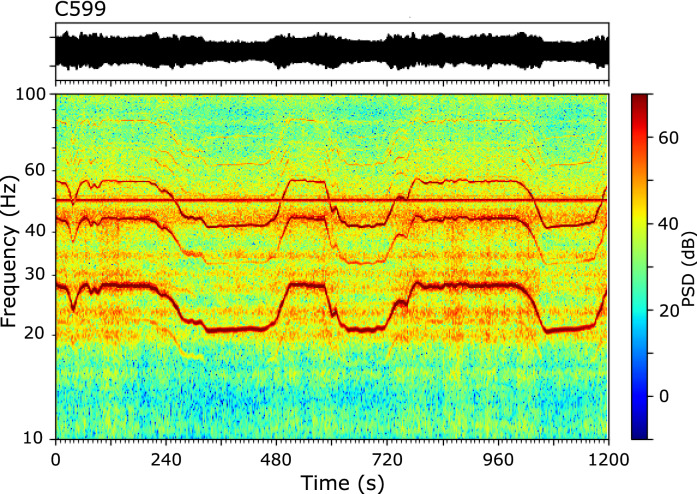
Seismic record and corresponding spectrogram for a 20 min long time interval at station C599. Large energy harmonics with varying frequency are related to the rotation of the WT blades, while the harmonic bands at constant frequencies are related to the oscillations of the WT tower.

The most elevated part of the Central System is equipped with a large number of wind turbines (WT) producing electrical power. More precisely, CIMDEF stations C599 and C600 were deployed at about 50 m of the basement of two different WT towers part of the Cabeza Mesá aeolian park (https://www.thewindpower.net/windfarm_es_9705_cabeza-mesa.php, last access October 2021). The wind-related signals in these two stations have a much larger amplitude than others and their spectral content is very different to that discussed previously. Figure 9 shows a 20 min interval record at station C599. Contrarily to the examples in Fig. 8c, the spectrogram shows the presence of multiple harmonics. The harmonic with largest amplitude has frequencies between 20 and 30 Hz, while the high order harmonics reach 120 Hz, the maximum frequency that can be analyzed by our data. Accordingly with^36^, we interpret that the signals recorded at these stations are generated by the motion of the WT following the blowing winds, with the harmonics being related to the blade pass frequency of its rotors. Time intervals with high frequency correspond to stronger winds and hence faster blade rotations. Our spectrogram shows also the presence of resonance frequencies appearing as horizontal bands, with increased energy during the intervals of faster blade rotation. Following the same authors, we interpret these harmonics at constant frequencies as generated by the oscillations of the WT tower itself.

Several studies have documented that WT generate soil vibrations, propagating mostly as Rayleigh surface waves^37,38^ that are typically detected at distances of 4–5 km^36^. Their amplitude, related to wind speed, is high enough to be used for seismic interferometry^39^, but has a negative effect on the performance of seismic networks^40^. It is interesting to note that these studies focus in the 1–10 Hz band, in contrast with our observations, that detects maximum amplitudes at frequencies above 20 Hz. This is probably related to the geophones used in our experiment, with a natural frequency of 10 Hz.

### Rain and thunders

**Figure 10:**
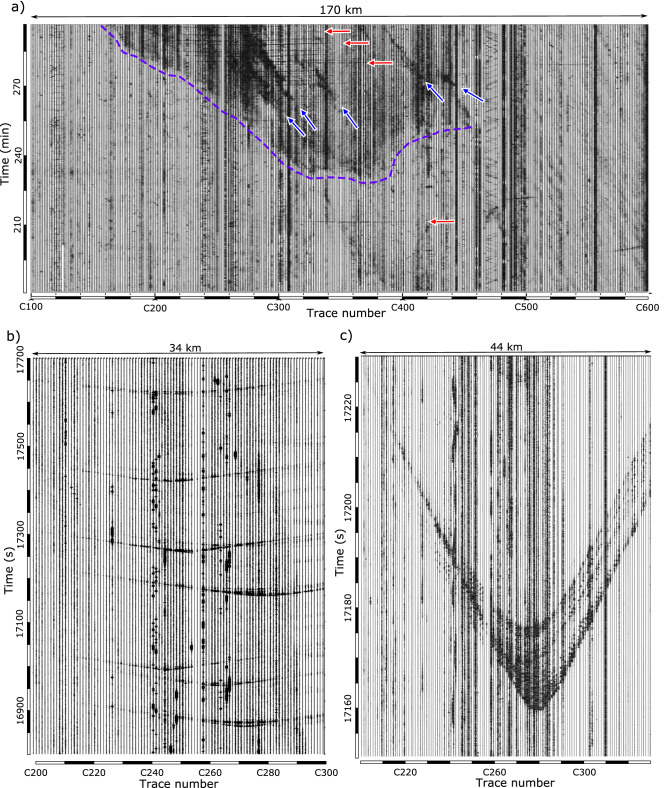
(**a**) Record section filtered between 70 and 90 Hz. Dashed blue line shows the area where a seismic amplitude increase associated with rainfall is detected. Blue arrows mark some of the signals related to wind gusts. Red arrows show some examples of signals related to thunders. (**b**) Selected section showing up to 7 events interpreted as due to thunders (**c**) Detail on the records of one of the thunder events. The distance range covered by each section is shown on top of the panels.

From the inspection of the entire record section, we can observe a clear increase in background noise at the end of the recording period, starting at traces C320-C380 and extending progressively to traces C180-C420 (Fig. 2). This high amplitude noise extends all around the spectra, but is clearer at high frequencies. Figure 10a shows the data recorded during the last 2 h after applying a 70–90 Hz band-pass filter which enhances this energy. The amplitude of this background seismic energy increase, as does its spectral content and its time evolution across the profile, strongly suggesting an origin related to rainfall. Seismic energy related to rainfall is a well-known feature, observed at different frequency ranges^41–43^ and seems mostly related to the impact of raindrops in the ground. The pattern of this noise in the record section shows the progression of the rainfall episode towards the north, as also shown by the presence of high energy bands starting at southern traces and progressing to the north. These later bands are interpreted as due to wind gusts associated with the rainfall.

During the same time interval, a number of short lasting, impulsive events appear in the record section as subhorizontal lines. Two of these events, separated by 3 min, can be identified in Fig. 2 around minute 154 between traces C370 and C465. Later on, a similar event is identified around minute 212, slightly before the arrival of the seismic energy generated by rainfall (traces C315–C410). In both cases, the signals extend along more than 30 km. During the last 30 min of recording, the time interval associated with heavier rain, these impulsive events appear more frequently, in particular in the trace range C220–C360. Figure 10b shows a blow-up of 7 of these events, showing that they propagate at acoustic speed and that they include different phases. These observations strongly suggest that the events are due to acoustic waves generated by thunders, coupling to mechanical waves near the seismometers. Seismic recordings of thunders are a well-known feature^44,45^, but they are usually recorded at isolated seismic stations, while in this case the high-density data available allows a detailed monitoring of the wave propagation.

Figure 10c shows the recording of one of these thunder-related events. The signal arrives first at trace C280, located 12 km west of the city of Zamora. The first arrival times are symmetric around an axe located at this trace, indicating that the origin was close to the seismic profile. However, the coda of the signal is very different at both sides, including several wave-trains in the southern part of the section but converging to a single signal to the north.

The seismic detection of acoustic events is a subject of increasing interest and evidences of signals related to thunders^46^, accidental industrial explosions^47,48^ or bolide explosions^49^ have been reported recently. We think that seismic data, in particular when acquired in dense networks, can be of great interest for atmospheric scientists, as they provide a new tool to investigate the atmospheric properties and monitor accurately the time evolution of storms, complementing tools as lightning location and meteorological radars. Interdisciplinary cooperation will be needed to take advantage of the data.

## Discussion and conclusions

The acquisition of controlled source seismic profiles using a densely spaced array of sensors not only allows to obtain high resolution models of the subsurface, but also gives the opportunity to record in great detail other natural and anthropogenic sources of noise. Using a 5-h long record acquired along 300 km by a high-density network composed of almost 1000 seismic stations, we have identified ground motions generated by different sources, some of them related to acoustic-mechanical wave coupling. The data inspection has also allowed us to pin-point a large quarry blast event, that can provide additional data to constrain the crustal models developed in the investigated area.

Particular attention has been given in this contribution to the long-range recording of signals generated by an airplane and a helicopter flying in opposite directions along the profile. The spectrograms of both signals allow to easily discriminate between both, due to the harmonics produced by the helicopter blade rotation. Our data allows to estimate the velocity and direction of each aircraft along distances of 150–200 km, with traces every 350 m, resulting in an exceptional dataset. CIMDEF seismic traces provide also detailed monitoring of road traffic. In areas with scarce vehicle displacements, signals related to individual vehicles can therefore be identified and followed over distances of several hundreds of meters. Close to heavier traffic roads, individual vehicles are difficult to identify in the trace wiggles, but appear more clearly in the spectrograms, allowing to estimate their speed using the Doppler effect formula, a fact that, to our knowledge, has not been previously described.

Records related to wind, rainfall and thunder events can provide the basis for an interdisciplinary collaboration with meteorological sciences. The detailed analysis of these different events may help to track in detail the storm evolution and to monitor the changes in atmospheric parameters affecting wave propagation.

As a summary, we are convinced that this contribution proves that the data acquired in the framework of wide-angle reflection and refraction seismic experiments can be useful beyond modelling of subsurface from the seismic waves generated by the controlled source, opening the door to investigate a range of multidisciplinary processes.

## Methods

Seismic wiggles and record section plots are produced using the ObsPy software^50,51^ and the Seismic Analysis Code (SAC)^52^.

Spectrograms were calculated using the ObsPy software^50,51^, with a window length of 20 s and a large overlapping (80%) allowing to smooth the image. A color palette, expressed in dB and relative to a reference value of 1 (m^2^/s^4^)/Hz, is used to show the energy distribution.

The velocity of the moving source is estimated, based on the Doppler effect, from the expression

$${\text{v}} = {\text{c}}\left( {{\text{F}}_{{\text{h}}} {-}{\text{F}}_{0} } \right)/{\text{F}}_{{\text{h}}}$$

where c is the sound speed, F_0_ is the real frequency of the source and F_h_ is the uppermost frequency observed. F_0_ corresponds to the value where the concavity of the signal changes. For the examples shown in Fig. 3b, F0 is 22.6 Hz and F_h_ has values of 26.4 and 26.8 Hz at stations C524 and C528 respectively. Assuming a sound speed of 345 m/s, the calculated velocities are 179 and 195 km/h.

The location of the quarry blast is done using the Hypo71 software (USGS: Hypo71PC original manual and binary, https://pubs.er.usgs.gov/publication/ofr75311), widely used in the seismological community.

## References

[1] Díaz J, Ruiz M, Sánchez-Pastor PS, Romero P (2017). Urban Seismology: on the origin of earth vibrations within a city. Sci. Rep..

[2] Lecocq T, Hicks SP, van Noten K, van Wijk K, Koelemeijer P (2020). Global quieting of high-frequency seismic noise due to COVID-19 pandemic lockdown measures. Science.

[3] Larose E (2015). Environmental seismology: What can we learn on earth surface processes with ambient noise?. J. Appl. Geophys..

[4] Díaz J (2016). On the origin of the signals observed across the seismic spectrum. Earth Sci. Rev..

[5] Wilkinson MD (2016). Comment: The FAIR Guiding Principles for scientific data management and stewardship. Sci. Data.

[6] Wilkinson MD (2016). The FAIR guiding principles for scientific data management and stewardship. Sci. Data.

[7] DeFelipe I (2021). Reassessing the lithosphere: SeisDARE, an open-access seismic data repository. Earth Syst. Sci. Data.

[8] Ayarza, P. & Carbonell, R. CIMDEF: a wide-angle deep seismic reflection profile in the Central Iberian Zone. 10.20350/digitalCSIC/10528 (2020).

[9] Palomeras I (2009). Nature of the lithosphere across the Variscan orogen of SW Iberia: dense wide-angle seismic reflection data. J. Geophys. Res. Solid Earth.

[10] Ehsan SA (2015). Lithospheric velocity model across the Southern Central Iberian Zone (Variscan Iberian Massif): The ALCUDIA wide-angle seismic reflection transect. Tectonics.

[11] Andrés J (2019). Lithospheric image of the Central Iberian Zone (Iberian Massif) using global-phase seismic interferometry. Solid Earth.

[12] Andrés J (2020). What can seismic noise tell us about the Alpine reactivation of the Iberian Massif? An example in the Iberian Central System. Solid Earth Discuss..

[13] De Felipe, I. *et al*. Crustal Imbrication in an Alpine Intraplate Mountain Range: a wide-angle cross-section across the Spanish-Portuguese Central System. Tectonics submitted, (2022).

[14] Rodríguez Fernández, L. R. *et al*. Mapa Geológico de España y Portugal a escala 1: 1.000.000. (2015).

[15] Jarvis, A., Reuter, H. I., Nelson, A. & Guevara, E. Hole-filled seamless SRTM data V4, International Centre for Tropical Agriculture (CIAT). https://srtm.csi.cgiar.org (2008).

[16] Damarla TR, Ufford D (2008). Helicopter detection using harmonics and seismic-acoustic coupling. Unattended Ground Sea Air Sens. Technol. Appl. X.

[17] Eibl EPS, Lokmer I, Bean CJ, Akerlie E, Vogfjörd KS (2015). Helicopter vs volcanic tremor: characteristic features of seismic harmonic tremor on volcanoes. J. Volcanol. Geotherm. Res..

[18] Meng H, Ben-zion Y (2018). Characteristics of airplanes and helicopters recorded by a dense seismic array near anza california. J. Geophys. Res. Solid Earth.

[19] Eibl EPS, Lokmer I, Bean CJ, Akerlie E (2017). Helicopter location and tracking using seismometer recordings. Geophys. J. Int..

[20] Meng H, Ben-Zion Y (2018). Characteristics of airplanes and helicopters recorded by a dense seismic array near anza California. J. Geophys. Res. Solid Earth.

[21] Quiros DA, Brown LD, Kim D (2016). Seismic interferometry of railroad induced ground motions: Body and surface wave imaging. Geophys. Suppl. Month. Not. R. Astron. Soc..

[22] Brenguier F (2019). Train traffic as a powerful noise source for monitoring active faults with seismic interferometry. Geophys. Res. Lett..

[23] Nakata N, Snieder R (2012). Estimating near-surface shear wave velocities in Japan by applying seismic interferometry to KiK-net data. J. Geophys. Res. Solid Earth.

[24] Behm M, Leahy GM, Snieder R (2014). Retrieval of local surface wave velocities from traffic noise – an example from the La Barge basin (Wyoming ). Geophys. Prospect..

[25] Schippkus S, Garden M, Bokelmann G (2020). Characteristics of the ambient seismic field on a large-N seismic array in the Vienna Basin. Seismol. Res. Lett..

[26] Meng H, Ben-Zion Y, Johnson CW (2021). Analysis of seismic signals generated by vehicle traffic with application to derivation of subsurface q-values. Seismol. Res. Lett..

[27] Chambers K (2020). Using DAS to investigate traffic patterns at Brady Hot Springs, Nevada, USA. Lead. Edge.

[28] Spica ZJ, Perton M, Martin ER, Beroza GC, Biondi B (2020). Urban seismic site characterization by fiber-optic seismology. J. Geophys. Res. Solid Earth.

[29] Yuan S, Lellouch A, Clapp RG, Biondi B (2020). Near-surface characterization using a roadside distributed acoustic sensing array. Lead. Edge.

[30] Fuchs F, Bokelmann G (2018). Equidistant spectral lines in train vibrations. Seismol. Res. Lett..

[31] Hirn A, Daignières M, Gallart J, Vadell M (1980). Explosion seismic sounding of throws and dips in the continental Moho. Geophys. Res. Lett..

[32] Carbonell R (1996). Crustal root beneath the urals: Wide-angle seismic evidence. Science.

[33] De Angelis S, Bodin P (2012). Watching the Wind: Seismic Data Contamination at Long Periods due to Atmospheric Pressure-Field-Induced Tilting. Bull. Seismol. Soc. Am..

[34] Lott FF, Ritter JRR, Al-qaryouti M, Corsmeier U (2017). On the analysis of wind-induced noise in seismological recordings. Pure Appl. Geophys..

[35] Withers MM, Aster RC, Young CJ, Chael EP (1996). High-frequency analysis of seismic background noise as a function of wind speed and shallow depth. Bull. Seismol. Soc. Am..

[36] Neuffer T, Kremers S, Fritschen R (2019). Characterization of seismic signals induced by the operation of wind turbines in North Rhine-Westphalia (NRW) Germany. J. Seismol..

[37] Stammler K, Ceranna L (2016). Influence of wind turbines on seismic records of the Gräfenberg array. Seismol. Res. Lett..

[38] Westwood RF, Styles P (2017). Assessing the seismic wavefield of a wind turbine using polarization analysis. Wind Energy.

[39] Friedrich T, Zieger T, Forbriger T, Ritter JRR (2018). Locating wind farms by seismic interferometry and migration. J. Seismolog..

[40] Neuffer T, Kremers S (2017). How wind turbines affect the performance of seismic monitoring stations and networks. Geophys. J. Int..

[41] Dean T (2017). The seismic signature of rain. Geophysics.

[42] Diaz J, Schimmel M, Ruiz M, Carbonell R (2020). Seismometers within cities: a tool to connect earth sciences and society. Front. Earth Sci..

[43] Nørmark, E. Wind and rain induced noise on reflection seismic data.10.3997/2214-4609.20144485 (2011).

[44] Kappus ME, Vernon FL (1990). The acoustic signature of thunder from seismic records. J. Acoust. Soc. Am..

[45] Lin TL, Langston CA (2007). Infrasound from thunder: A natural seismic source. Geophys. Res. Lett..

[46] Zhu T, Stensrud DJ (2019). Characterizing thunder-induced ground motions using fiber-optic distributed acoustic sensing array. J. Geophys. Res. Atmos..

[47] Schneider FM (2018). Seismo-acoustic signals of the Baumgarten (Austria) gas explosion detected by the AlpArray seismic network. Earth Planet. Sci. Lett..

[48] Fuchs F, Schneider FM, Kolínský P, Serafin S, Bokelmann G (2019). Rich observations of local and regional infrasound phases made by the AlpArray seismic network after refinery explosion. Sci. Rep..

[49] Hedlin MAH, Drob D, Walker K, Hedlin CDG (2010). A study of acoustic propagation from a large bolide in the atmosphere with a dense seismic network. J. Geophys. Res. Solid Earth.

[50] Krischer L (2015). ObsPy: a bridge for seismology into the scientific Python ecosystem. Comput. Sci. Discov..

[51] Megies T, Beyreuther M, Barsch R, Krischer L, Wassermann J (2011). ObsPy - what can it do for data centers and observatories?. Ann. Geophys..

[52] Goldstein, P., Dodge, D., Firpo, M. & Lee, M. SAC2000: Signal processing and analysis tools for seismologists and engineers Title. in The IASPEI International Handbook of Earthquake and Engineering Seismology (eds. Lee, W., Knamori, H., Jennings, P. & Kisslinger, C.) (Academic Press, 2003).

